# Five-gene signature associating with Gleason score serve as novel biomarkers for identifying early recurring events and contributing to early diagnosis for Prostate Adenocarcinoma

**DOI:** 10.7150/jca.52170

**Published:** 2021-04-30

**Authors:** Lingyu Zhang, Yu Li, Xuchu Wang, Ying Ping, Danhua Wang, Ying Cao, Yibei Dai, Weiwei Liu, Zhihua Tao

**Affiliations:** 1 Department of Laboratory Medicine, The Second Affiliated Hospital of Zhejiang University School of Medicine, Hangzhou, 310009, China.; 2 Department of Biochemistry and Molecular Biology, Bengbu Medical College, Anhui 233030, China.

**Keywords:** prostate cancer, gene signatures, robust rank aggregation, Weighted Gene Co-expression Network Analysis, disease-free interval, inflammation landscape

## Abstract

**Background:** Compared to non-recurrent type, recurrent prostate adenocarcinoma (PCa) is highly fatal, and significantly shortens the survival time of affected patients. Early and accurate laboratory diagnosis is particularly important in identifying patients at high risk of recurrence, necessary for additional systemic intervention. We aimed to develop efficient and accurate diagnostic and prognostic biomarkers for new PCa following radical therapy.

**Methods:** We identified differentially expressed genes (DEGs) and clinicopathological data of PCa patients from Gene Expression Omnibus (GEO) datasets and The Cancer Genome Atlas (TCGA) repositories. We then uncovered the most relevant clinical traits and genes modules associated with PCa prognosis using the Weighted gene correlation network analysis (WGCNA). Univariate Cox regression analysis and multivariate Cox proportional hazards (Cox-PH) models were performed to identify candidate gene signatures related to Disease-Free Interval (DFI). Data for internal and external cohorts were utilized to test and validate the accuracy and clinical utility of the prognostic models.

**Results:** We constructed and validated an accurate and reliable model for predicting the prognosis of PCa using 5 Gleason score-associated gene signatures (ZNF695, CENPA, TROAP, BIRC5 and KIF20A). The ROC and Kaplan-Meier analysis revealed the model was highly accurate in diagnosing and predicting the recurrence and metastases of PCa. The accuracy of the model was validated using the calibration curves based on internal TCGA cohort and external GEO cohort. Using the model, patients could be prognostically stratified in to various groups including TNM classification and Gleason score. Multivariate analysis revealed the model could independently predict the prognosis of PCa patients and its utility was superior to that of clinicopathological characteristics. In addition, we fund the expression of the 5 gene signatures strongly and positively correlated with tumor purity but negatively correlated with infiltration CD8+ T cells to the tumor microenvironment.

**Conclusions:** A 5 gene signatures can accurately be used in the diagnosis and prediction of PCa prognosis. Thus this can guide the treatment and management prostate adenocarcinoma.

## Introduction

Prostate adenocarcinoma (PCa) is the second most prevalent malignant tumor in men worldwide, accounting for about 15% of all tumors in males. In Europe and the United States, for the first time, the incidence of prostate cancer stands at 1.051% and continues to rise 1. Although the incidence of prostate cancer in China is lower than that in the United States, it is gradually increasing and remarkably varies among races in the country. In 2018, there were 120,000 cases of PCa in China. Besides these bleak statistics, PCa cases are projected to rise to 172,000 in 2022 2. Over the past few decades, prostate-specific antigens (PSA) have been used as effective biomarkers for diagnosing and monitoring prognosis of PCa patients. Combined with imaging, the diagnosis efficiency of PCa has substantially improved. The diagnosis and treatment module of prostate cancer has dramatically changed following the identification and widespread use of PSA for screening. However, there are still limitations that reduce its effectiveness, especially at a lower concentration (4-10 ng/mL) 3-5.

Advances in chip technology and next-generation high-throughput sequencing (NGS) have facilitated the identification of several novel biomarkers in tumor tissues, serum, and even urine. Substantial reports have implicated nucleic acid molecules such as mRNAs, ncRNAs (miRNAs, lncRNAs or circRNAs) and membrane proteins present in extracellular vesicles (EVs) including exosomes, microvesicles and microparticles in the pathogenesis and progression in PCa 6-10. However, only a few biomarkers have been approved for use by the US Food and Drug Administration (FDA) (PSA in 1994, PHI in 2012, and PCA3 in 2012). Ideal diagnostic biomarkers should be highly specific (correctly identify populations without specific diseases; true negative rates) and sensitive (correctly identify groups with specific diseases; true positive rates), easy to use, reproducible, easy to acquire and quantify and easy to interpret across different centers. Thus accurate biomarkers that can stratify patients in to different PCa prognosis risk groups can guide the development and application of targeted interventions depending on treatment response. This underlines the urgent need for accurate biomarkers for diagnosis and monitoring of PCa response to chemotherapy.

Identification of aberrant gene expression is an effective method of identifying biomarkers because it assesses tumor activity and expression profiles of several genes in separate tissue types. Thus multiple cancer subtypes can be distinguished based on gene expression. Molecular features of tumors can potentially inform on prognosis of the cancers. Even though both proteins and RNA inform on molecular activities, detection and quantification of RNA is often easier, even in trace amounts and complex matrix environment. Furthermore, the multiplicity of RNA-based assays is fairly simple, which implies that thousands of potential targets can be evaluated simultaneously using high-throughput assays. RNA biomarkers have been discovered through analysis of ncRNAs, multi-gene expression panels, alterations in the presence of splice variants, and gene fusion transcripts, premised on various tumor cell functions. Most primary stage of PCa are almost asymptomatic, and only few of them are detected through randomized physical examinations. Thus most cancers advance to worse stages before related signs begin to emerge, limiting treatment efficacy and survival time of respective patients. Intriguingly, most patients with PCa do not die from tumors at the primary tumor, but rather metastases related complications in other distant organs, despite an initially high complete optimal response rate. Increasing evidence suggests that only a quarter of patients with metastatic and invasive PCa can survive longer than 5 years after an initial diagnosis of the tumors 11-13. Notably, the majority of invasive prostate cancer advances into metastatic disease after local treatment, leaving PCa as an incurable and fatal malignancies after local treatment. Thus metastatic PCa, particularly the castration-resistant prostate cancer (CRPC), accounts for about two-thirds of prostate cancer mortalities 12, 14, 15.

Despite the high prevalence of PCa, accurate diagnostic and prognostic biomarkers for the tumor are still lacking. PSA concentrations have been used in routine screening of PCa. However, the technique is confusing for PSA concentrations between 4 ng/mL and 10 ng/mL. Unfortunately, up to 11% of men present with PSA concentrations lower than 2.0 ng/mL, thus can easily be missed using the PSA technique only 11. The ratio free PSA (fPSA) to total PSA (tPSA) effectively enhances the diagnostic sensitivity and specificity of PCA technique. However, the serum based PSA technique cannot stratify PCa patients in to prognosis risk groups 16-18. The second generation of genome analytical technologies such as microarray analysis and NGS have deepened our understanding on the cellular and molecular mechanisms underlying PCa. This has presented unique opportunities to explore large genomic data, with a view of discovering novel biomarkers for various disease parameters. Thus the low cost and accuracy presented by the new technologies have opened a new frontier in to personalized genomic diagnosis and clinical management. As one of the most prominent themes in cancer genetics, the characteristic changes in the somatic genome of tumor tissue are valuable parameters in diagnosing and predicting treatment response of tumors. The currently available methods include analysis of genetic signatures in peripheral blood and characterization of DNA/RNA in tumor cells (CTCs) 19-23. Despite the advances in tumor genetics, the molecular basis for occurrence and prognosis of PCa remains to be validated. The genetic pathways and/or gene expression profiles associated with chemotherapy response and prognosis of PCa represent a promising opportunity for unraveling the above relationships.

Early diagnosis and prognosis prediction have invaluable benefits to the patients, underlining the need for accurate and reliable PCa associated biomarkers. In this study, we analyzed integrated PCa cases with disease-free interval (DFI) data from two independent cohort studies (TCGA-PRAD and GSE116918) to develop and validate novel personalized genetic signatures associated with development and prognosis of PCa. We also investigated clinical and pathological features and immune infiltration landscape in PCa tissues. Identification of diagnostic and prognostic-related genes for PCa patients at an early stage will unravel the complex molecular mechanism between gene expression and PCa tumorigenesis and progression.

## Materials and methods

### Selection of PCa gene expression datasets

The GEO microarray datasets utilized in this study were downloaded from the Gene Expression Omnibus (GEO) repository (http://www.ncbi.nlm.nih.gov/) 24. To be included in this study, the dataset must have fulfilled the following: (1) contained expression profile of both prostate cancer tissue and corresponding adjacent normal prostate tissue, excluding benign proliferative prostate tissue, (2) was of satisfactory sample size (Normal sample ≥6, Tumor sample ≥6), (3) contained complete probe names (or probe sequences) and corresponding gene symbols (raw probes numbers ≥20000). Accordingly, GSE3325 25, GSE6956 26, GSE32448 27, GSE3251 28, GSE46602 29, GSE55945 30, GSE34312 31, GSE69223 32, GSE71016 33, GSE88808 34 fulfilled the inclusion criteria, and were therefore included in the subsequent analyses. PCa RNA-sequencing profiles and clinical data were extracted from the TCGA database (https://cancergenome.nih.gov/).

### Identification of DEGs

We identified DEGs using the Robust Rank Aggregation (RRA) method. The DGEs were cleaned and analyzed using potential probability models, based on Bonferroni corrected P values were used in identifying significant differential gene expression to minimize false positive results 35. The data was further cleaned and normalized using the “limma” package in R-software before importation in to RRA for further meta-analyses 36.

### Gene function enrichment analyses

The functional pathways regulated by key DGEs were identified using “Clusterprofiler” package in R software 37, following Gene Ontology (GO) enrichment and Kyoto Encyclopedia of Genes and Genomes (KEGG) pathway enrichment analyses. Statistical significance for the pathways in PCa was set at P-value < 0.05 and false discovery rate (FDR) < 0.05. The expressing of DEGs between normal and PCa tissues was plotted using the “Clusterprofiler” packages. Notably, this study only highlighted the molecular function (MF), biological processes (BP), and cell composition (CC) most influenced by the DEGs.

### WGCNA analysis

Gene expression patterns in multiple samples were assessed using WGCNA. Gene modules that regulate physiological process or different tissues processes were further investigated to unravel their clinical significance 38. The expression profiles and clinical parameters of these DEGs were extracted from the TCGA data portal, and subsequently merged for subsequent analyses. Overall, we incorporated 7 clinicopathological parameters: T grade, N grade, M grade, Diagnosis age, Recurrence status, Plymphnode number, and Gleason score. The relationship between weighted gene co-expression modules and integrated clinical traits was evaluated using the WGCNA platform in R package. In the WGCNA algorithm, the elements in the defined gene co-expression matrix were the weighted values of the correlation coefficients of the genes were the key elements in the defined gene co-expression matrix. Significance was set at scale-free topological fitting index (R^2^ = 0.96). In the clustering tree, genes with high absolute correlation were assembled into the invariable co-expression module. A cluster tree was then generated based on FlashClust analysis. Genes were divided into distinct gene modules based on TOM-based difference metrics, based on the association between modules and clinical traits. The module Gene Significance (GS) and Module Membership (MM) were calculated to identify significant gene modules. The relationship between the gene modules and the 7 clinicopathological parameters was visualized using a heat map, at P-value < 0.05. The biological functions of modules with the strongest association with clinical traits were further evaluated through GO and KEGG analyses.

### DFI associated biomarkers

Based on TCGA database, new locoregional recurrences, distant metastases and new primary tumors were all classified in to new tumor events. DFI was defined as period between the initial follow-up to the appearance of a new event. We then used the TCGA-PRAD data (Prostate adenocarcinoma) to validate the association between the most significant DEGs and DFI. Univariate Cox regression analysis identified 113 significant DEGs (P-value < 0.05), which were included in the subsequent analyses. Lasso regression analysis was performed to identify the most significant prognosis-related differently expressed gene signatures 39-41. The optimal lambda was identified after running 1,000 stimulations based on cross-validation likelihood. Overall, 5 genes were identified, which were subsequently included in the multivariate Cox-PH regression model. Using the default coefficients in the multivariate Cox regression analyses, the risk score prediction model based on gene signature expression is set by a linear combination of expression levels of independent gene signatures. The risk-factors scoring algorithm was generated for prognosis prediction was as follows: Risk-score = Σ (βmRNA × exprmRNA). Something needs to be noted that βmRNA denotes the Cox-PH coefficient of mRNA while exprmRNA denotes the mRNA expression levels. Using the risk scoring prediction model, PCa patients were subdivided into low-risk and high-risk groups based on the median risk score. The DFI of low-risk patients and high-risk patients were calculated using the Kaplan-Meier survival curves and compared using the log-rank test. Time-dependent ROCs (Receiver Operating Characteristic Curves) were used to assess the predictive efficiency of PCa prognosis model, whereas the area under the curve (AUC) of the ROC was used to estimate the prediction accuracy of the model. The predictive nomogram incorporated all significant independent predictors identified in the multivariate Cox-PH regression model 42. Risk curves and scatter plots were generated to display the relationship between the risk score and the PCa patient factors included in the TCGA. The robustness of the prognostic model was validated using the GSE116918 dataset 43.

### External validation of the expression levels of 5 gene signatures

The relevance of the gene expression signature on prognosis and clinicopathological factors was verified using the TCGA dataset and the protein expression pattern was verified in Human Protein Atlas 44. The differential expression of the 5 signature genes in primary prostate cancer and metastatic prostate cancer tissues was analyzed using the GSE32269 dataset 45. The difference in gene expression between prostate cancer and normal prostate tissue was analyzed based on ROC. The cutoff threshold was based on the maximized of Youden's index (Sensitivity + Specificity -1). Statistical significance was set at P<0.05. All the statistical tests were two-sided.

### Gene set enrichment analysis

The differential gene expression between high-risk and low-risk group patients was assessed based on GSEA. Statistical significance was set at P < 0.05 and FDR < 0.05. The function of the top 8 most differently expressed genes in the high-risk and low-risk groups were analyzed using the GO and KEGG gene sets.

### Patients and clinical tissue samples

Clinical tissue samples for 15 pairs of freshly snap-frozen prostate cancer tissues and paired normal adjacent tissues were histopathologically and clinically diagnosed at The First Affiliated Hospital of Bengbu Medical College. All patients included in this study consented to the use their tissue samples and associated demographical and clinical data in a de-identified format in this research. The protocol for this study was approved by the Ethical Examination committee of Biomedical Research in People (Trial) (2007) and the Institutional Ethical Review Board of Anhui Cancer Hospital.

### Immunohistochemical staining

After fixation in formalin, the clinical pathological tissues were quickly frozen to -25 °C and cut in to thin 4-μm sections are produced. Immunohistochemistry tests were performed as previously described/following the manufacturer's protocol. Briefly, the sections were t firsts incubated at 4 °C, overnight with primary antibodies (ZNF695, 10508-1-AP, Proteintech; CENPA, 26754-1-AP, Proteintech; TROAP, 15911-1-AP, Proteintech; BIRC5, 13634-1-AP, Proteintech; KIF20A, 25556-1-AP, Proteintech) in a blocking solution. Second incubation was performed using peroxidase-coupled IgG (Burlingame, CA). The immunofluorescences were visualized after staining and counterstaining with diaminobenzidine and hematoxylin, respectively.

### Validation of mRNA expression for the 5 gene signatures

Total RNA in the tissues was extracted from tissues using TRIzol reagent, according to the manufacturer's protocol (Invitrogen, California, USA). The RNA quality was assessed based on A260/A280 ratios using an ND-1000 UV Nanodrop spectrophotometer (Thermo Scientific). Then RNAs were then reverse transcribed into cDNA by using the reverse transcription kit (TaKaRa, Japan) for mRNA expression. The quantitative reverse transcriptase PCR (qRT-PCR) was performed using the SYBR premix Ex Taq-II kit (Takara, Japan), in an ABI 7500HT fast real-time PCR System (Applied Biosystems, USA). GAPDH was used as the internal control. The relative mRNA expression was determined based on the comparative cycle threshold (2-ΔΔCt) equation. The primer sequences used in this research are listed in Supplementary Table S1.

### The relationships between expression of the gene signatures and immune microenvironment

We used CIBERSORT (http://cibersort.stanford.edu/) 46 and TIMER (https://cistrome.shinyapps.io/timer/) 47 datasets (two genes expression-based deconvolution algorithm) to analyze the infiltration of immune cells in tumor tissues relative to normal tissues based on mRNA seq data. CIBERSORT contains data for 22 different immune cells including monocytes, NK T cells, B cells, T cells, and so on. On the other hand, TIMER repository contains data for 6 immune infiltrates (B cells, CD4+ T cells, CD8+ T cells, Neutrophils, Macrophages, and Dendritic cells).

### Statistical analysis

Unless otherwise indicated, continuous data was expressed as mean ± standard error of the mean (SEM). Differences between two groups were analyzed using an unpaired Student's t-test. Two-way t-test or paired t-tests were used for groups with normally distributed data but with different variance; otherwise, a two-sided Wilcoxon test was used. Multiple hypothesis tests were performed using the Benjamini and Hochberg methods unless otherwise stated. Statistical analysis was performed using RStudio software v1.2.1335 (RStudio Inc.) and GraphPad Prism software v8.0 (GraphPad Software Inc.). Statistical significance was set at P < 0.05.

## Results

### Incorporating GEO datasets and screening DEGs using RRA method

**Figure 1:**
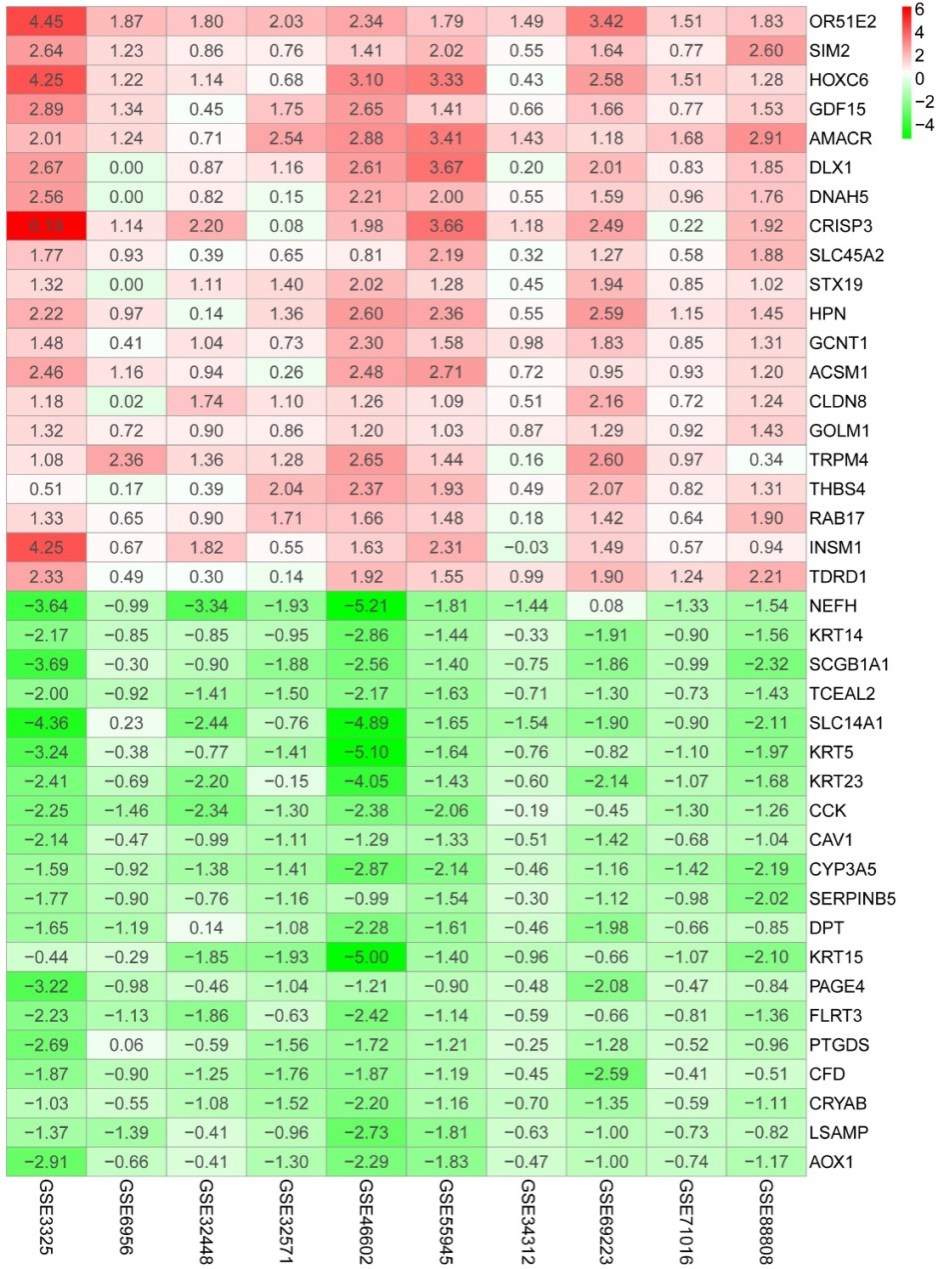
Heatmap shows top 20 DEGs in up-regulated and down-regulated genes based on RRA DEGs were defined with P-value < 0.05 and |logFC| ≥ 1.

**Figure 2:**
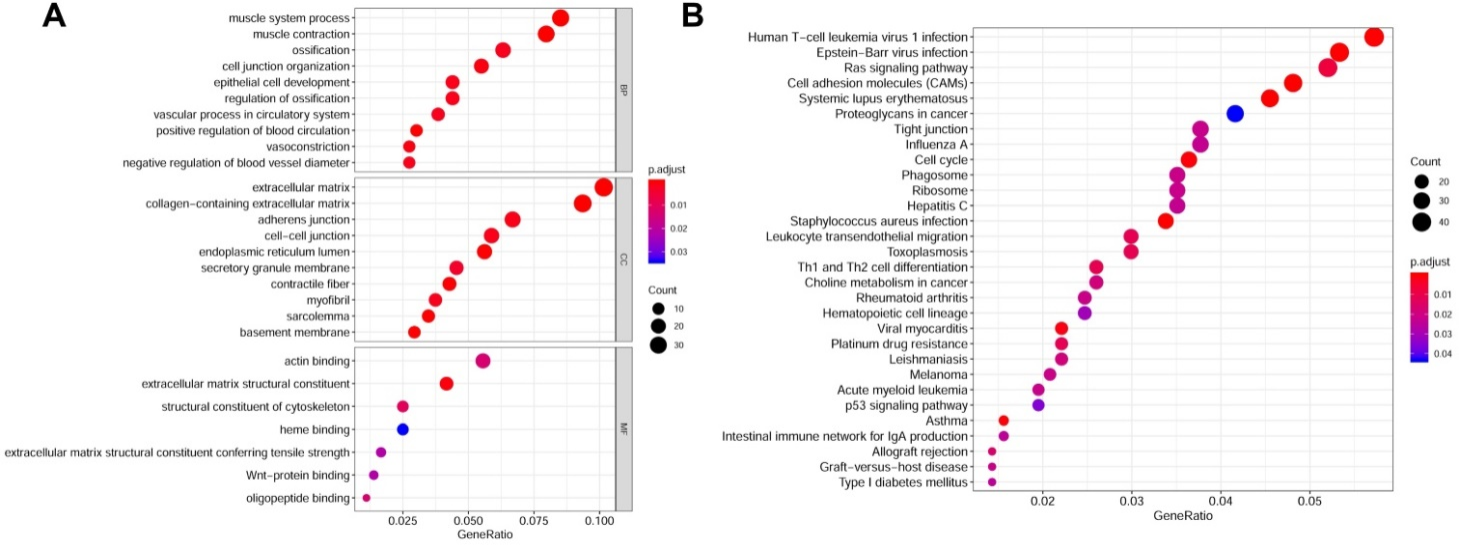
Gene functions enrichment analyses of DEGs. **(A)** GO terms enrichment analysis of DEGs in MF, BP, and CC. **(B)** KEGG pathways enrichment analysis of DEGs.

The flow diagram for the development and validation of the 5 gene prognostic model for PCa is shown in **Figure S1**, following RRA analysis of 10 microarray datasets extracted from the GEO repository. Relevant characteristics of these datasets such as ID, platform, the number of raw probes, and the number of samples are shown in **Table 1**. Raw data for each GEO dataset were first standardized to the baseline data of non-carriers following conversion to z scores (**Figure S2A-J**). RRA analysis identified 1128 up-regulated genes and 962 down-regulated DEGs. The top 20 up-regulated and down-regulated DEGs based on logFC values are shown in **Figure 1**. The biological functions of the top 400 DEGs (200 up-regulated genes and 200 down-regulated genes) were identified following GO and KEGG analyses. Based on GO analysis, the genes regulated several molecular functions (MF), biological processes (BP), and cell composition (CC) (**Figure 2A**). In particular, the model genes regulated muscle system process, extracellular matrix, and the actin-binding. On the other hand, KEGG pathway analysis revealed that the DEGs were significantly enriched during human T-cell leukemia virus 1 infection and Epstein-Barr virus infection as well under activated Ras signaling pathway, etc. (**Figure 2B**).

### WGCNA analysis and Key module identification

**Figure 3:**
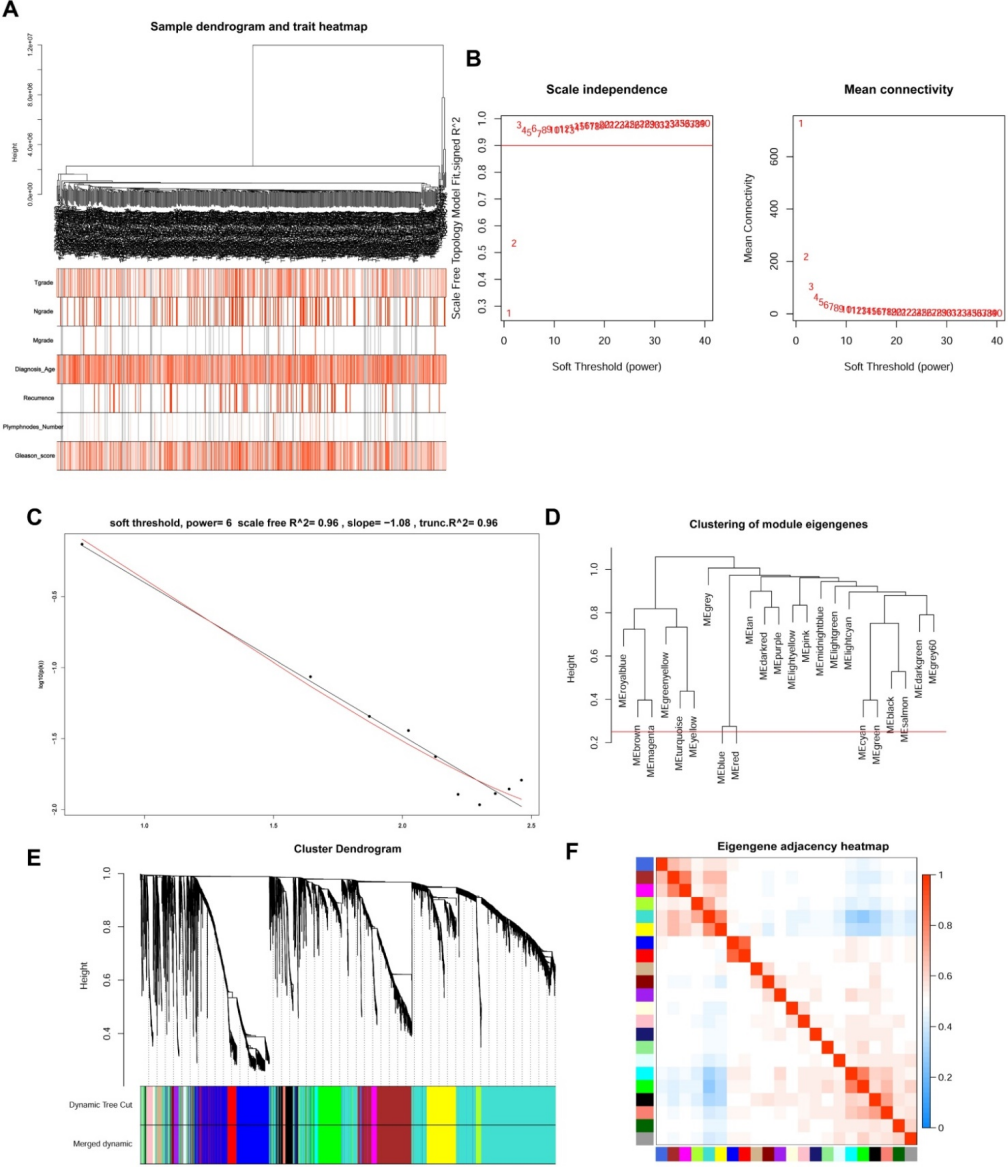
WGCNA analysis for DEGs. **(A)** Dendrogram and traits heatmap between TCGA-PRAD samples and clinical traits. **(B)** Analysis of the average connectivity and scale-free fit index by setting unequal soft-thresholding power. **(C)** The scale-free R^2^ reached its maximum value when setting soft-thresholding power at 6. **(D)** Clustering of module eigengenes. The red line indicates cut height (0.20). **(E)** Dendrogram of all DEGs clustered based on TOM. **(F)** Analysis of the relationship of co-expression modules based on the pearson correlation coefficient.

**Figure 4:**
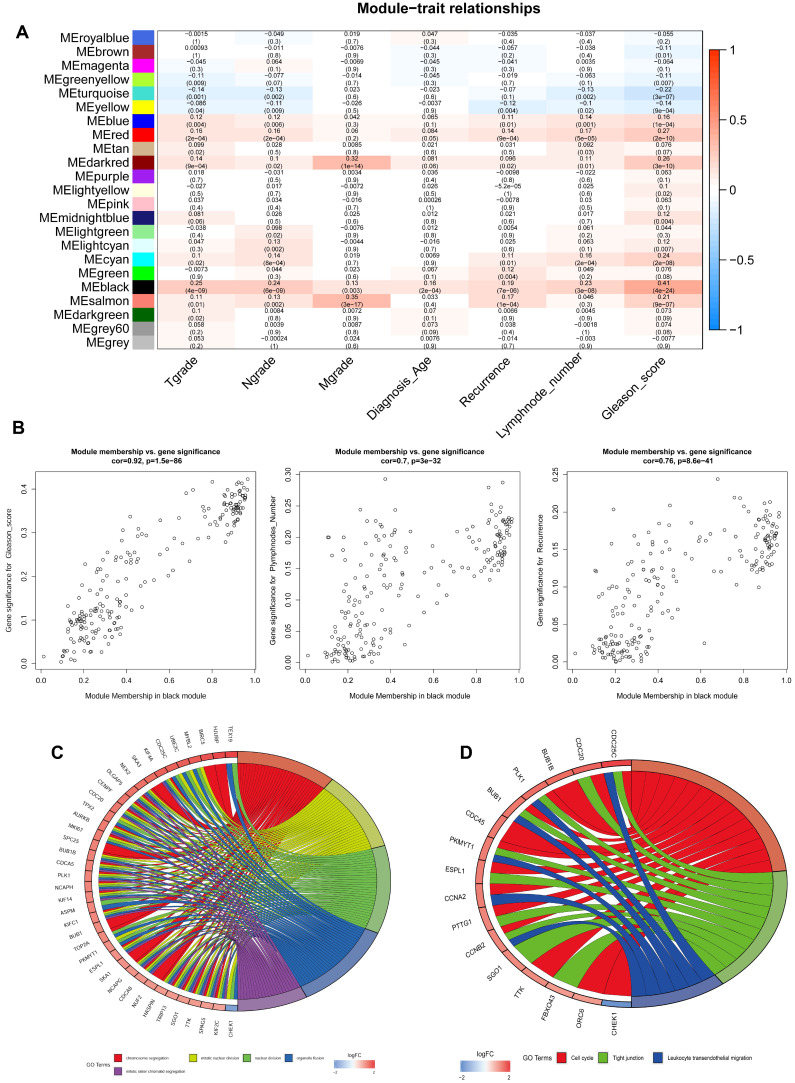
Identification of key genes and functional annotation of the black module. **(A)** The correlation between modules and the clinical traits. **(B)** Scatter plot of module eigengenes in the black module related with Plymphnodes Number (correlation coefficient = 0.7), Recurrence (correlation coefficient = 0.76), and Gleason score (correlation coefficient = 0.92). **(C)** Chord plot depicted the relationship between genes and GO terms of molecular function. **(D)** Chord plot indicated the relationship between genes and KEGG pathways.

The top 5000 up-regulated genes based on RRA analysis subjected to WGCNA analysis. To determine key modules for DEGs and clinical traits of PCa patients, we merged clinical traits and expression profiles of prostate cancer from TCGA. The clinical traits included TNM grade, age at diagnosis, recurrence status, number of metastatic lymph nodes, and Gleason score. Dendrogram and traits heatmap for the PCa patients are shown in **Figure 3A**. Based on scale-free R^2^ = 0.96 (**Figure 3B-C**) and cutting height of 0.20 (**Figure 3D**), we identified the gene co-expression modules as shown in **Figure 3E**. Heat map for the interactions of these co-expression modules (**Figure 3F**) revealed that the expression black module strongly and positively correlated with the Gleason score (correlation coefficient = 0.41, P = 4E-24) (**Figure 4A**). Interestingly, a scatter plot of gene significance (GS) and module membership (MM) of the black module genes revealed that MM in the black module significantly correlated with Plymphnodes Number (correlation coefficient = 0.7, P = 3e-32), Recurrence (correlation coefficient = 0.76, P = 8.6e-41), and Gleason score (correlation coefficient = 0.92, P = 1.5e-86) (**Figure 4B**). After dropping 227 genes from the black module, GO and KEGG analyses revealed the remaining genes participated in chromosome segregation, mitotic nuclear division, nuclear division, organelle fission and mitotic sister chromatid segregation-22 (**Figure 4C**). KEGG pathway analysis on its part revealed the genes regulated the Cell cycle, Tight junction, and Leukocyte transendothelial migration (**Figure 4D**).

### Genes associated with DFI

**Figure 5:**
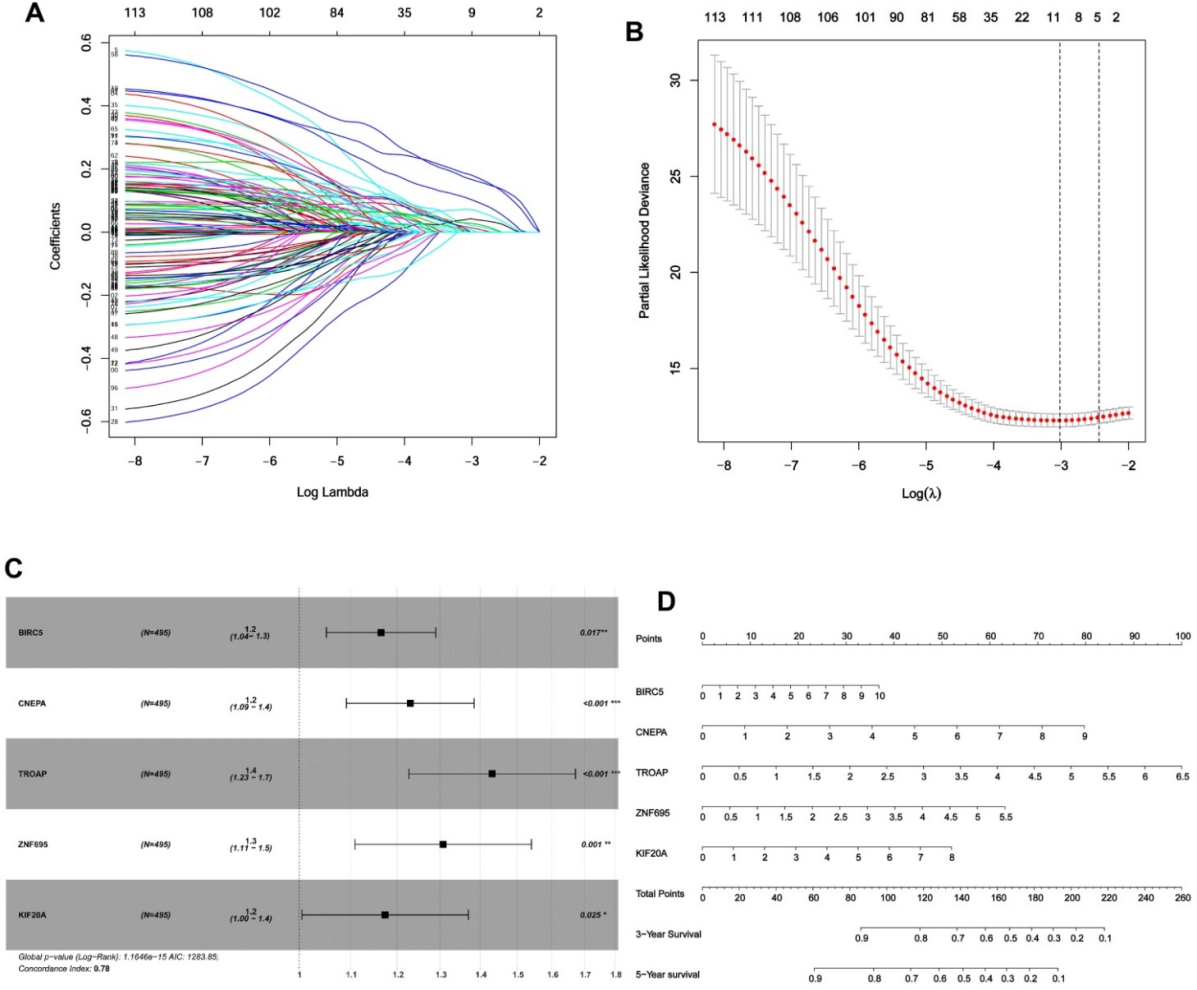
Identification of gene signatures associated with disease-free interval based on Cox-PH regression model. **(A)** Lasso coefficient profiles of the 495 progression- associated events in PCa. **(B)** Selection of the tuning parameter (λ) in the LASSO model through 10-fold cross-validation procedure was plotted as a function of log(λ). **(C-D)** Construction of multivariate Cox-PH regression model and ZNF695, CENPA, TROAP, BIRC5, KIF20A were considered significant and used to construct a prognostic model.

**Figure 6:**
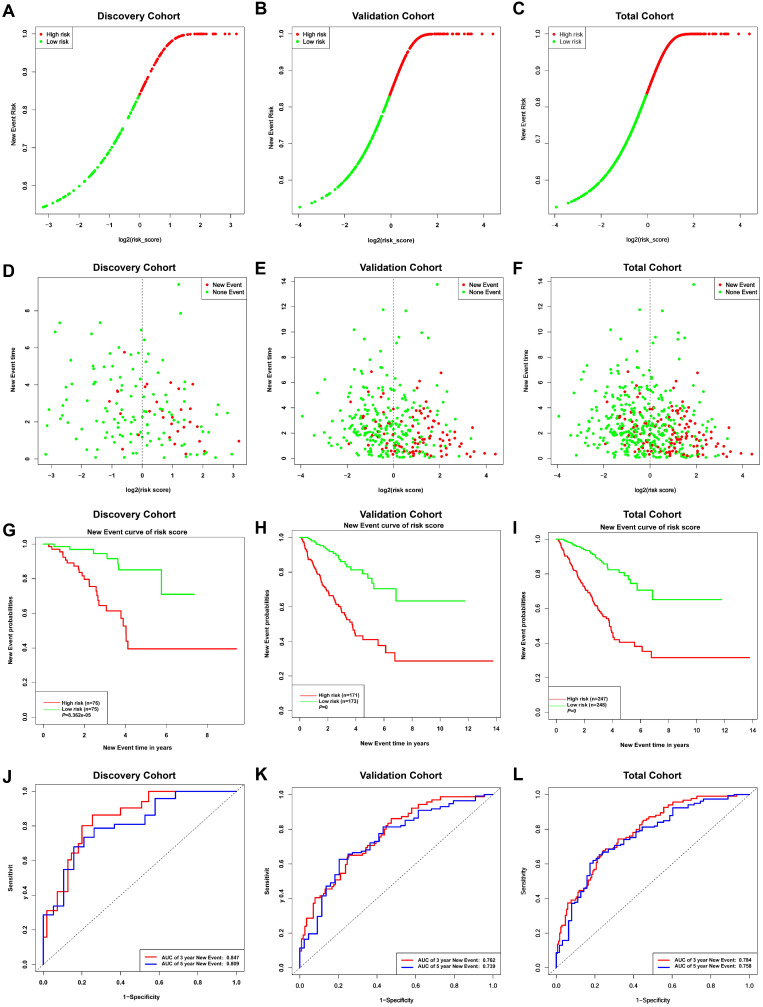
Risk curves **(A-C)** and scatter plots **(D-F)** implied the risk score and new event risk for each PCa patient. **(G-I)** KM survival curves revealed that the prediction model of risk score had good discrimination and patients with high-risk scores have a shorter disease-free interval. **(J-L)** According to the prognostic model, the ROC curve has a higher efficiency in predicting 3 or 5 years of DFI in PCa patients.

Given the results in **Figure 4**, we speculated expression of black module genes may impact on PCa outcome (Gleason score, recurrence, lymph node metastasis, etc.). We filtered the gene pool to obtain fewer candidate variants. In the end, we remained with 113 genes that were included in the lasso regression model. According to the results of Cross-validation for tuning parameter selection in the proportional hazards model, 5 genes were incorporated into the multivariate Cox regression model (**Figure 5A-B**). The multivariate regression analysis and the hazard ratio (HR) of these 5 genes was based on the risk score at 95% confidence interval (95% CI) is shown in **Figure 5C** (Concordance Index=0.78). The constructed nomogram incorporating expression profile of hub genes (log2 transform) for prognosis of PCa is shown in **Figure 5D**. The prognostic model was evaluated using expression profile of genes in 495 PCa tissues extracted from TCGA database. The data was randomly classified into the discovery cohort and the validation cohort in the ratio of 3:7. The Cox-PH model based on 5 gene signatures was executed to further validated the robustness of the prognostic model using the discovery cohort, validation cohort, and total cohort. Scatter plots were generated to display the risk score and new event risk of PCa patients in the total cohort. The clinicopathological characteristics of patients in the total cohort are shown in **Table 2**. Patients in high-risk groups were more likely to develop new events than their low-risk group counterparts (**Figure 6A-F**). Then, the Kaplan-Meier curve revealed that the accuracy and specificity of the risk score based prediction model was satisfactory (P < 0.001), with patients in the high-risk score group found to have a shorter disease-free interval (**Figure 6G-I**). The AUC curve for 3 or 5 year DFI of the PCa patients was 0.784, and 0.758, respectively (**Figure 6J-L**). Overall, these findings demonstrated the independent prognosis value of the 5 gene signatures (ZNF695, CENPA, TROAP, BIRC5 and KIF20A) in PCa.

### 5 Gleason score-associated gene signatures are independent risk factors for tumor recurrence in PRAD

**Figure 7:**
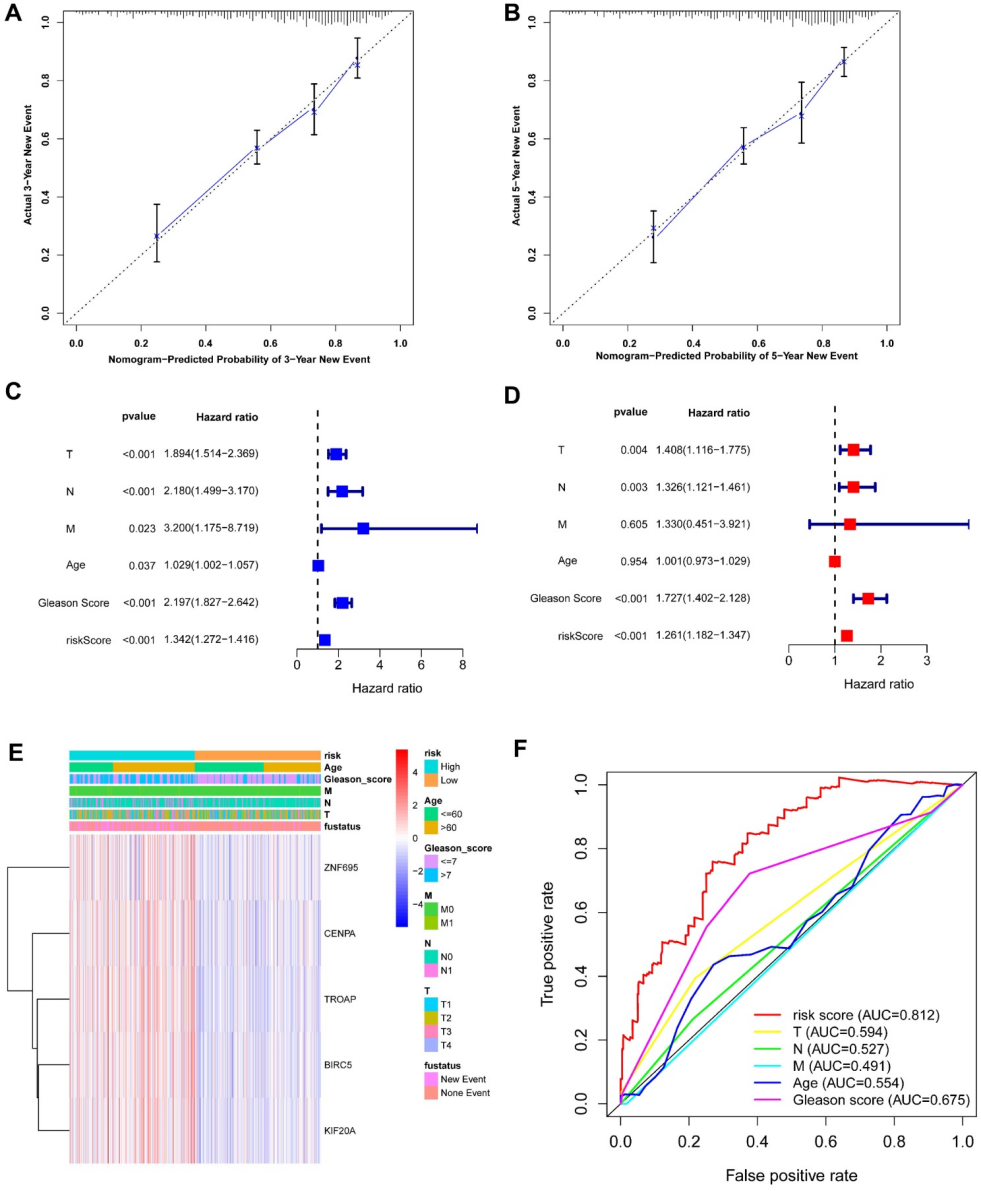
** (A-B)** Calibration curves of the nomogram for predicting the probability of DFI at 3 and 5 year. **(C-D)** The univariate and multivariate Cox regression analysis of risk score, age, Gleason score, and TNM stage. **e** Distribution characteristics of expression profiles of 5 gene signatures in different risk and clinicopathological groups. **(F)** Multiline ROC curves showed the superiority of 5-gene panel based on a 10-fold cross-validation, for predicting new tumor events.

**Figure 8:**
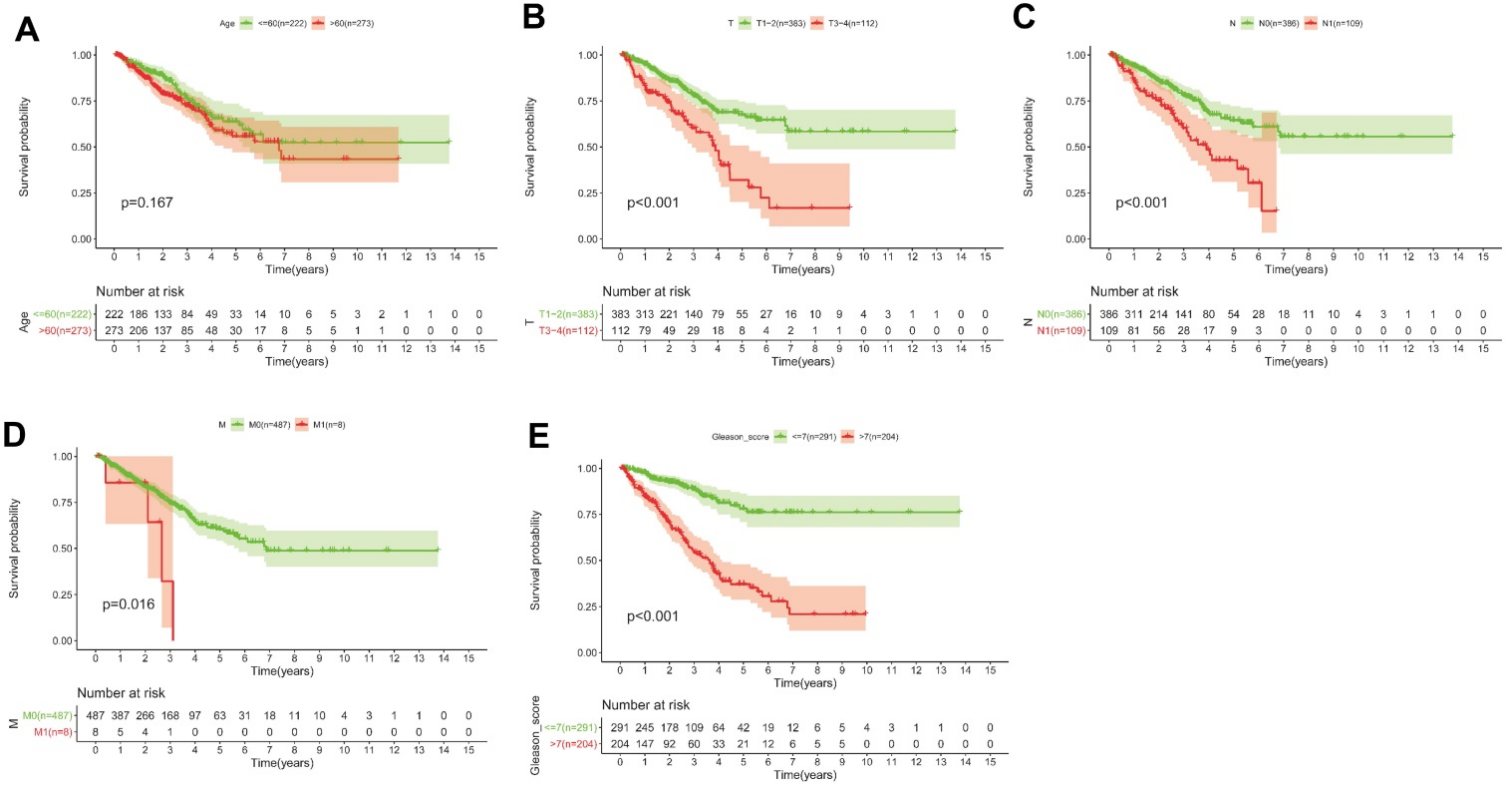
** (A-E)** KM survival curves shows that T classification (P < 0.001), N classification (P < 0.001), M classification (P = 0.016) and Gleason score (P < 0.001) are independent risk factors, except for age (P = 0.167).

**Figure 9:**
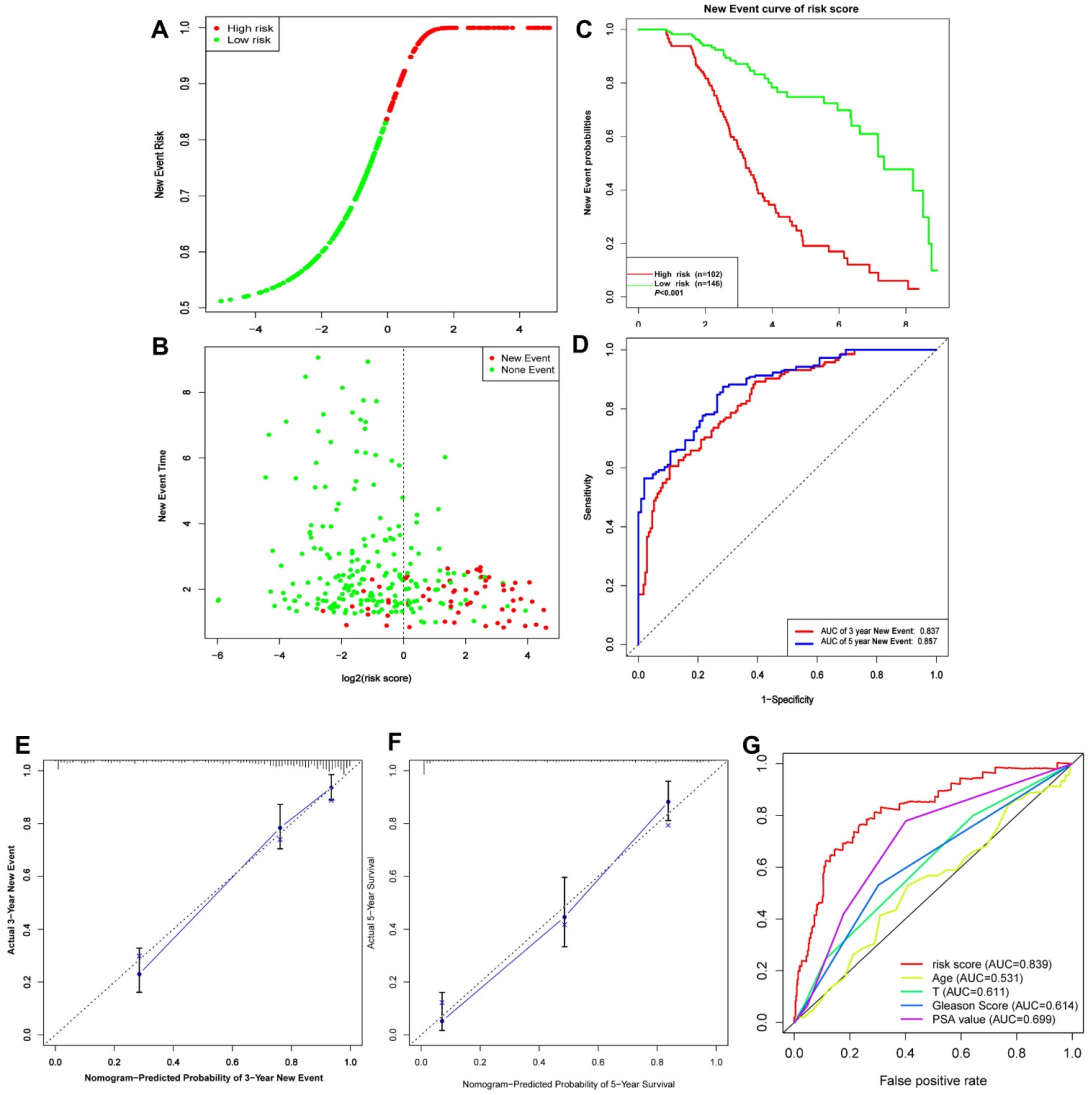
** (A-D)** Risk score, KM survival curves and time-dependent ROC curves of DFI in GSE116918 validation cohort. **(E-F)** Calibration curves of the nomogram for predicting the probability of DFI at 3 and 5 year **(G)** Multiline ROC curves showed the superiority of 5-gene panel than Age, T classification, Gleason score, and PSA value.

**Figure 10:**
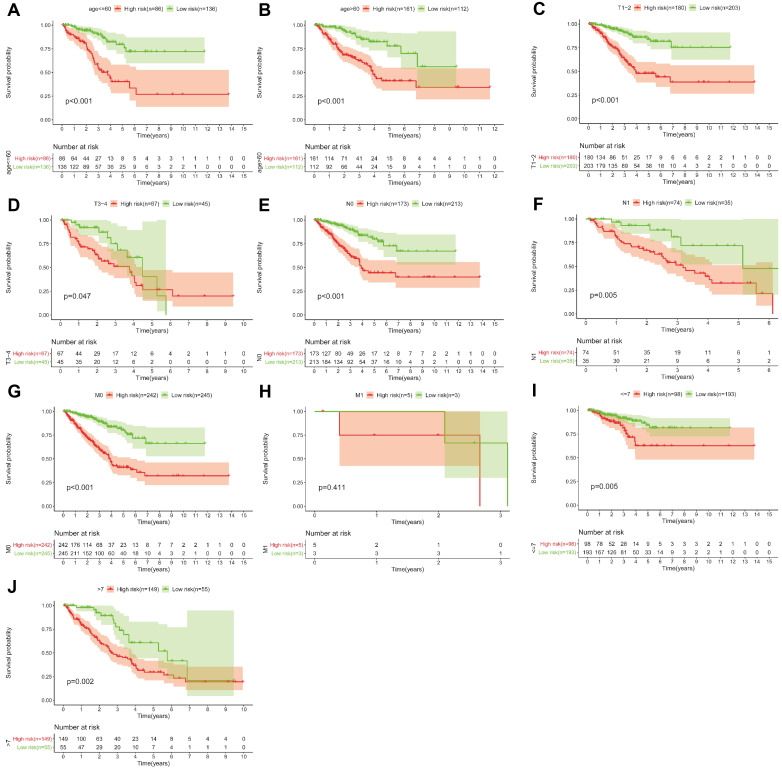
KM survival curves for the high and low risk groups stratified by clinicopathological variables. Age **(A, B)**, T classification** (C, D)**, N classification **(E, F)**, M classification **(G, H)**, and Gleason score **(I, J)**.

Based on the above-mentioned prediction model, the plotted calibration curve further demonstrated the strong consistency between predicted risk and the eventual outcome (**Figure 7A-B**). Various clinical variables affect the prognosis of PCa patients. Except for patient's Age, the other clinical characteristics (T, N, M, Stage, cancer status, and new tumor event) were all significantly associated with poor prognosis. Complete clinical characteristics carried by the TCGA-PRAD data set in **Table 2**. The correlation between different risk stratification and clinicopathological characteristics according to risk score was calculated using the chi-square test. However, forest plots for univariate and multivariate cox regression analysis revealed that the risk score could not only independently predict the prognosis of PCa, but its prediction power was superior to that of other clinical characteristics (**Figure 7C-D, F**). The distribution of clinicopathological characteristics and expression of gene signatures in low-risk and high-risk groups is displayed in **Figure 7E**. KM-plot revealed that compared to T1-2, patients in the T3-4 group displayed significantly shorter disease-free interval (P < 0.001) (**Figure 8B**). Similarly, patients in higher pathological grades have shorter disease-free interval, relative to the lower-grade counterparts (N1 vs N0, P < 0.001; M1 vs M0, P = 0.016; Gleason >7 vs Gleason ≤7, P < 0.001) (**Figure 8C-F**). However, we found age was not an independent predictor of PCa prognosis (**Figure 8A**). The accuracy, sensitivity and specificity of the novel model were validated using external GEO cohort (GSE116918) (248 samples). Based on the TCGA median risk score, the patients were divided into low-risk (n = 146) and high-risk groups (n = 102) (**Figure 9A-B**). K-M survival analysis revealed that compared to high-risk group patients, low-risk patients displayed longer disease-free interval (**Figure 9C**). Time-independent ROC curve and calibration curves further demonstrated the high robustness of the prediction model with regard to new tumor events after 3 (AUC = 0.837) or 5 years (AUC = 0.857) (**Figure 9D-F**). Survival age cluster analysis using the entire TCGA-PRAD cohort data revealed that compared to patients ≤60, those above 60 exhibited considerably lower disease-free interval (**Figure 10A-B**). All high risk group patients based on TNM or Gleason scores (**Figure 10C-J**) had a significantly shorter DFI relative to their low risk counterparts.

### Biological mechanisms underlying the function of model genes

**Figure 11:**
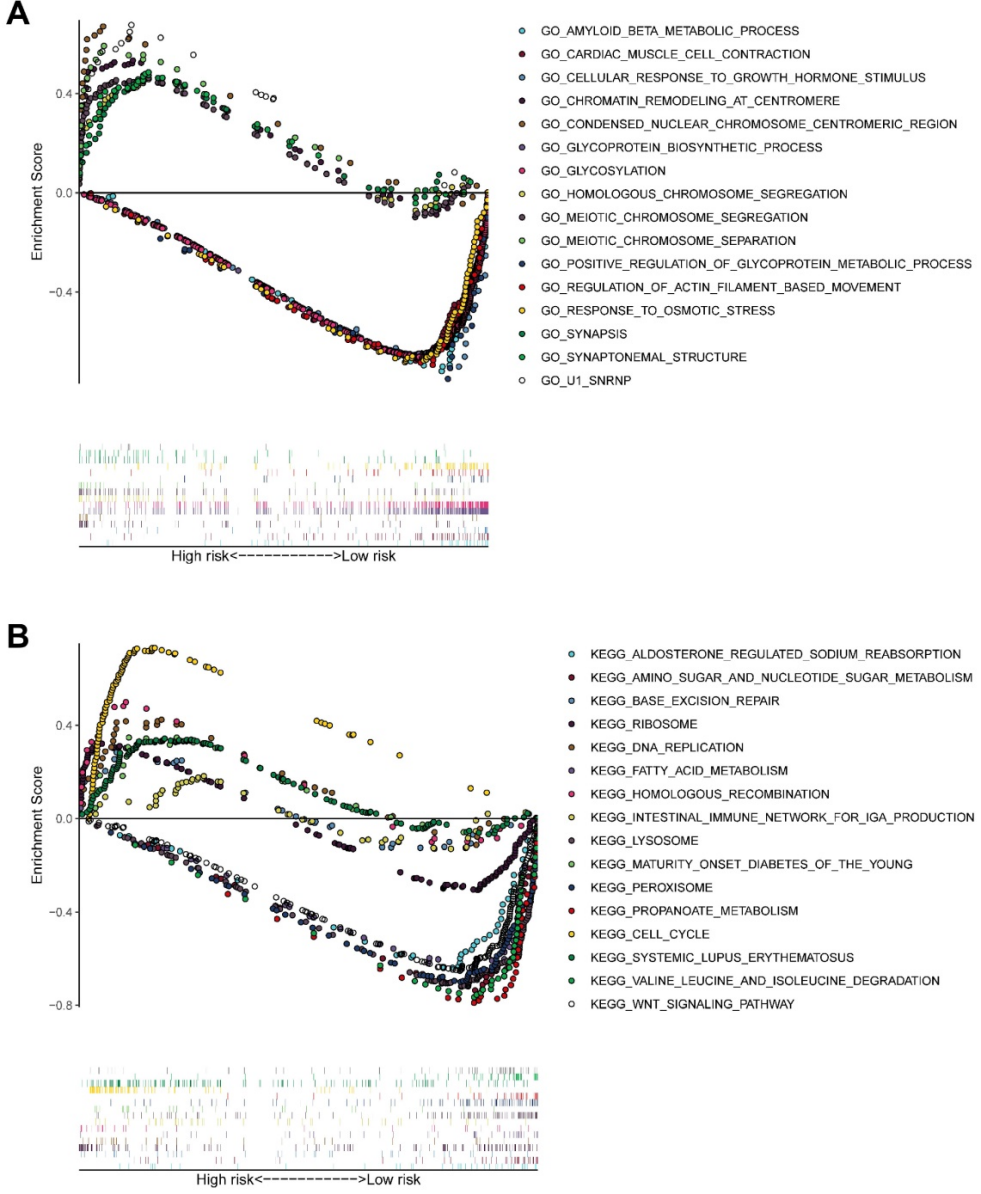
GSEA delineates biological pathways and processes between high and low risk using gene sets of GO **(A)** and KEGG **(B)**. Each run was performed with 1000 permutations.

GSEA (**Figure 11A-B**) of pathways identified following GO and KEGG analysis revealed that the novel model genes were over-expressed in the high-risk group individuals, and regulated multiple biological processes, such as meiotic chromosome segregation, chromatin remodeling at centromere, homologous chromosome segregation, cell cycle, homologous recombination, DNA replication, etc.

### Validation of 5 gene signatures in PCa

**Figure 12:**
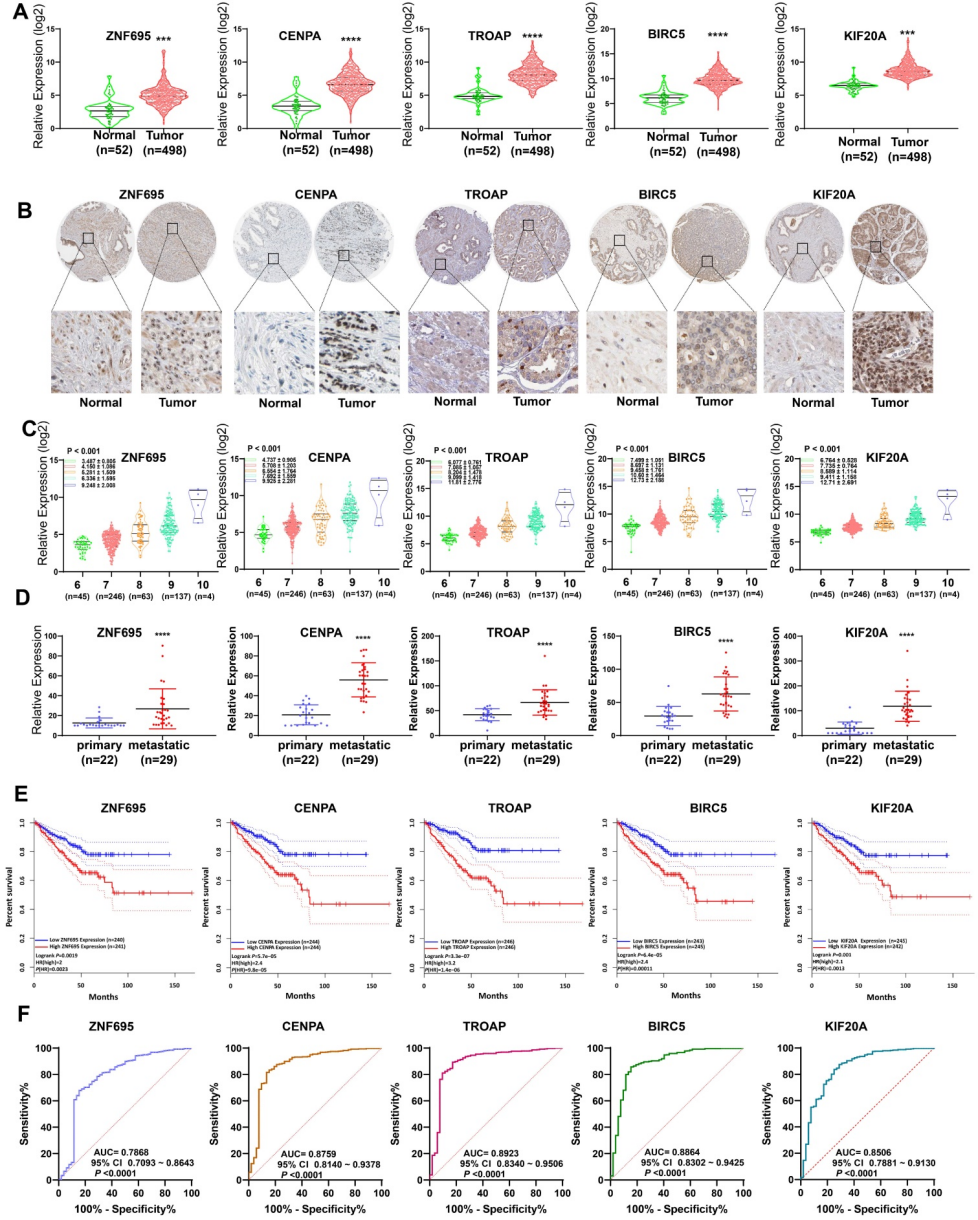
External validation of 5 gene signatures in PCa. **(A, B)** Differential expression of these 5 gene signatures in PCa tissue and paracancerous normal samples at mRNA and protein level.** (C)** The expression levels of these 5 gene signatures increased with increasing Gleason score. **(D)** The expression levels of these 5 gene signatures in primary and metastatic tumors. **(E)** Kaplan-Meier survival curves showed that higher expression of these genes was significantly associated with poor PFS. **(F)** ROC curve implied higher diagnostic efficiency of these 5 gene signatures.

In assessing the relationship between expression profiles of the model genes in PCa tissues and related clinicopathological characteristics based on TCGA data, we further found the 5 model genes were over-expressed in prostate cancer tissues, relative to normal prostate tissue (**Figure 12A**). Analysis of the Human Protein Atlas further validated the over-expression of proteins coded by the 5 signature gene in PCa tissues relative to paracancerous normal tissues (**Figure 12B**). Combined with WGCNA findings, it can be inferred that the expression of the 5 signature gene strongly and positively correlates with PCa Gleason score (**Figure 12C**). The expression levels of these 5 gene signatures in primary and metastatic tumors (bone metastasis or lymph node metastasis) were evaluated using GSE32269 data, also from GEO repository. Intriguingly, we found the five signature genes were over-expressed in metastatic tumors (**Figure 12D**), suggesting that up-regulated expression of the 5 signature genes promotes metastasis. Meanwhile, further revealed higher expression of the genes was strongly associated with shorter disease-free interval (**Figure 12E**). Moreover, the AUC plots demonstrated the expression pattern of the model genes was highly accurate in diagnosing PCa (**Figure 12F**). Based on Yuden index, the 5 model were highly specific and sensitive in predicting PCa prognosis (**Table 3**). Combined, AUC for the specificity and sensitivity of the 5 signature genes reached 0.9473 (95% CI = 0.9149 ~ 9698) (**Figure S3**).

### Expression of the 5 prognostic gene signatures in clinical samples

**Figure 13:**
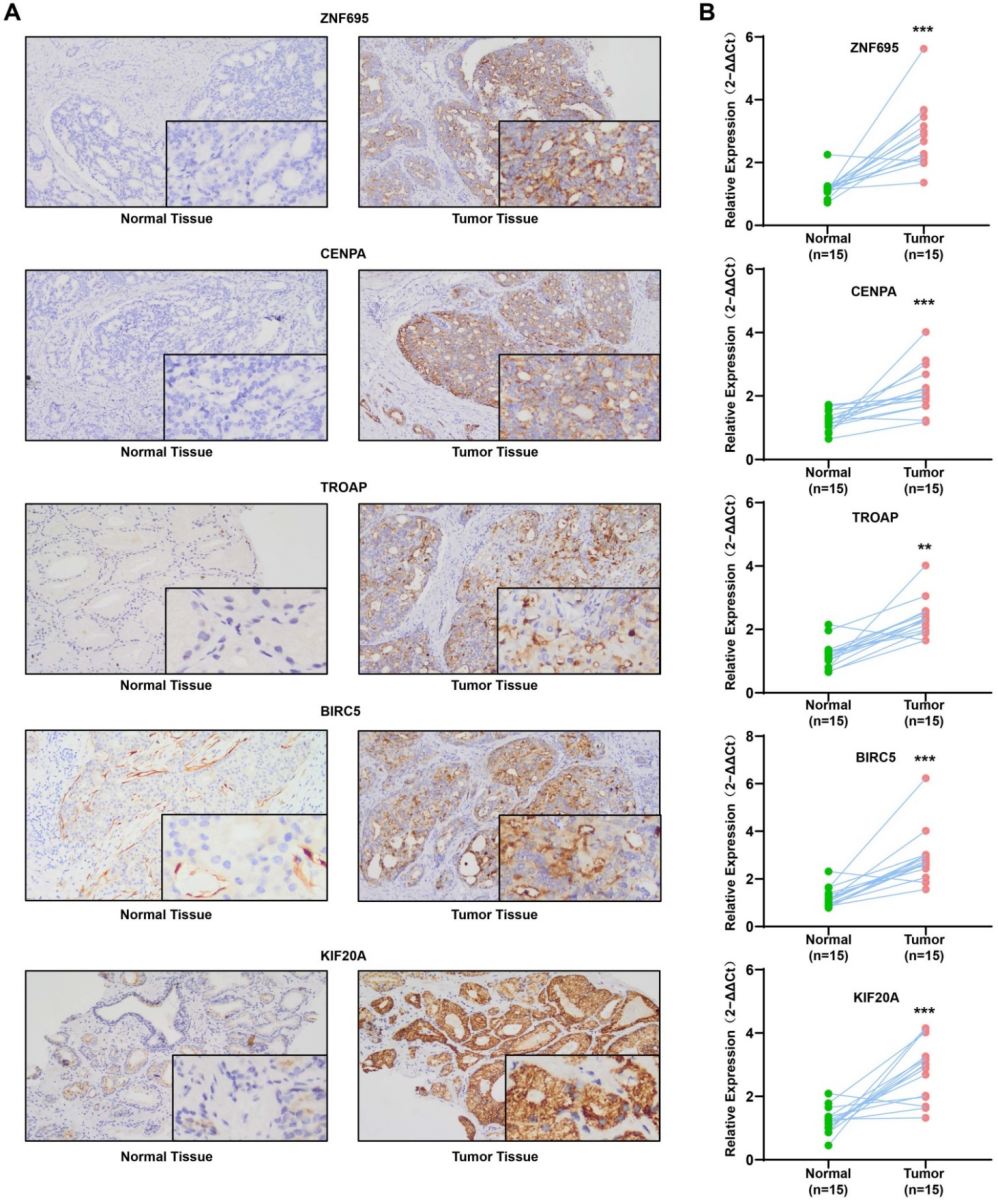
Experimental verification for 5 gene signatures. (A) IHC analysis was conducted to study altered protein expression in PRAD and paracancerous tissues (B) Increased expression of 5 genes was validated by qRT-PCR (**P < 0.01, ***P < 0.001).

QRT-PCR revealed that the mRNAs for the expression of the 5 signature genes (ZNF695, CENPA, TROAP, BIRC5, and KIF20A) were over-expressed in 15 PCa and adjacent tissues, relative to adjacent normal tissues (**Figure 13A**). Immunohistochemical (IHC) analysis further demonstrated moderate or high staining intensity of the 5 proteins in PCa tissues, relative to normal tissues (**Figure 13B**). These findings demonstrated the upregulated expression of signature gene in PCa tissues, consistent with previous bioinformatics analysis.

### Expression of signature genes and infiltration of immune cells in tumor tissues

**Figure 14:**
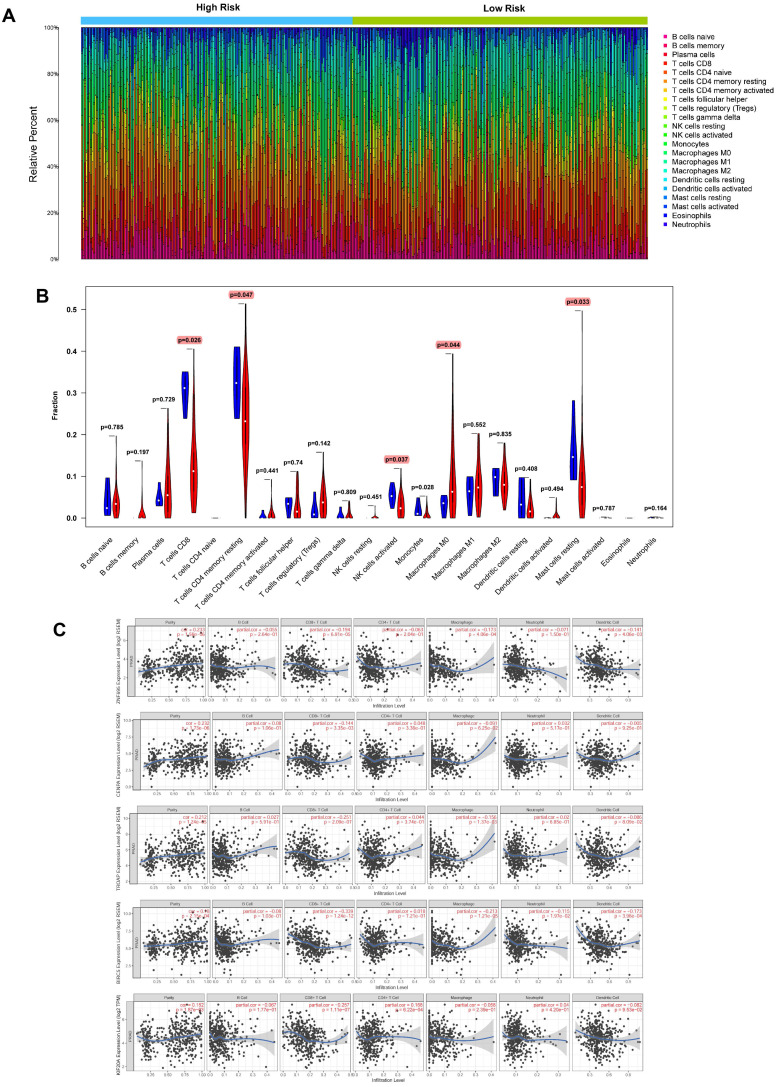
Evaluation of relationships between gene signatures and the immune microenvironment. **(A)** Assessment of the proportion of 22 immune cells calculated by the CIBERSORT algorithm in PCa tissues based on normalized expression in TCGA-PRAD. **(B)** Violin plot depicted the infiltration of significantly different subsetst (T cells CD8, T cells CD4 memory resting, NK T cells activated, Macrophages M0, and Mast cells resting) between the high and low risk. **(C)** TIMER algorithm implied these 5 gene signatures were positively correlated with tumor purity and negatively correlated with CD8+ T cells.

Based on CIBERSORT and TIMER analyses (**Figure 14A**) of TCGA-PRAD, we revealed strong exhaustion of T cells CD8 (P = 0.026), T cells CD4 memory resting (P = 0.047), NK T cells activated (0.037), Macrophages M0 (P = 0.044), and Mast cells resting (P = 0.033) (**Figure 14B**) in tissues of high-risk group patients, relative to their low-risk group counterparts. TIMER tool further revealed expression of the 5 signature genes (ZNF695, CENPA, TROAP, BIRC5 and KIF20A) was strongly and positively correlated with high tumor purity but negatively influenced the infiltration of CD8+ T cells and macrophages. In addition, we observed weak or no associations between the expression of the 5 genes and infiltration of B cells, CD4+ T cells, neutrophils, and dendritic cells (**Figure 14C**).

## Discussion

Prostate adenocarcinoma, the third most prevalent cancer globally, causes substantial morbidity and mortality in Europe and the United States. In China, PCa is among the top 10 causes of all disease morbidity and mortalities in the country. The increase in incidences of PCa has been attributed to the increase in older population and unhealthy diet 48-50. Despite the advances in molecular basis of PCa tumorigenesis, early diagnosis and prognosis prediction of PCa, particularly tumor recurrence, remain a challenge. The heterogeneous nature of PCa results in varied pathogenesis, and subsequently, diverse chemotherapy response. Numerous microarrays and RNA-seq studies have revealed effective therapeutic targets for PCa. Therefore, we aimed to identify novel genomic markers that assess PCa responses to different therapeutic interventions and subsequent prognosis. Overall, our univariate and multivariate Cox proportional hazard regression identified 5 key gene signatures (ZNF695, CENPA, TROAP, BIRC5 and KIF20A). WGCNA and hierarchical clustering analysis revealed that the DEGs between PCa and adjacent normal tissues were associated with several clinicopathological characteristics. In addition, ROC curves revealed that the signature model genes were accurate, highly sensitive and specific diagnosis biomarkers for PCa. Thus expression profile of these genes can be utilized clinically in assessing chemotherapy response of PCa with a view of improving the disease treatment outcome.

ZNF695 encodes the zinc finger family of proteins, whose function is not known, particularly in prostate cancer 51. In this study, we found ZNF695 was overexpressed in PCa, which correlated with worse disease outcomes such as tumor progression and metastasis. CENPA, a histone H3 variant, regulates chromosome segregation during cell division 52, 53. Over-expression of CENPA is associated with shorter DFS (Disease-free survival) in patients with breast cancer 54, whereas up-regulated CENPA-mediated pRb depletion promotes the development and progression of retinoblastoma 55. Meanwhile, research shows that TROAP is dysregulated in various tumors such as breast cancer, liver cancer, prostate cancer, and gastric cancer. The protein promotes proliferation and distant metastasis of tumors through multiple signaling pathways such as WNT3/survivin 56-60. In our study, we found TROAP promoted PCa metastasis, besides being an independent predictor of risk for new prostate cancer events. BIRC5 (Survivin) proteins perform multiple cell functions. In normal cells, they directly regulate apoptosis and filament-division of embryonic cells during embryo development. On the other hand, they promote tumor occurrence and metastasis of cancers. The expression of BIRC5 proteins correlates with poor differentiation and worse disease prognosis of malignant peripheral nerve sheath tumors, renal cell carcinoma, lung adenocarcinoma and ovarian cancer 61-64. Kinesin family member 20A (KIF20A) is a mitochondrial-related kinesin (MCAK) and is the most common member of the kinesin-6 proteins. It participates in disaggregation of microtubules, bipolar spindle formation, and chromosome segregation, and overall, mitosis and the cell-cycle 65, 66. Under or inhibition of KIF20A expression will disrupt the normal mitotic process, all of these are regarded as potential causes of tumorigenesis. The role of KIF20A in numerous tumors has been previously reported, such as bladder cancer, lung adenocarcinoma, renal clear cell carcinoma has been previously reported 67-70.

Our research proposes and validates a novel 5 gene signature that stratifies and predicts the prognosis of PCa patients. The prognostic biomarkers may guide clinical personalized treatment, potentially improving the disease outcomes. A wealth of compelling evidence demonstrates that tumor microenvironment influences progression of the cancer. Microenvironmental changes may further impact on tumorigenesis and relapse after chemotherapy. The majority of prostate cancers spread to the bones. Meanwhile, 65%-80% of prostate cancers advance to more aggressive forms 71, 72. The complex interactions between the skeletal microenvironment and tumor cells have been implicated in the development of castration-resistant prostate cancer (CRPC). Besides being stem cell precursors, bone marrow contains different types of recirculating mature immune cells, including Dendritic cells (DC), macrophages, different subsets of T and B lymphocyte subsets, myeloid-derived suppressor cells (MDSCs), and NK cells. Some of these leukocytes participate in the pathogen clearance and anti-tumor processes 73, 74. Using CIBERSORT and TIMER, we found a significant infiltration inhibition of several immune cell subsets, particularly CD8+ T cells, in PCa. Intriguingly, this phenomenon increased the risk of recurrence and metastasis. Given its immunomodulatory function, infiltration of immune cells in tumor micro-environment improves the overall survival of patients with varied tumor types 75-77. We found abnormal expression of the 5 signature genes inhibited optimal expression of CD8+ T cells in PCa tissues, particularly in high risk patients. Given the critical role of tumor immune microenvironment on metastasis, proper and timely interventions to dysregulated immune cell infiltration can avert tumor recurrence.

Besides PCa, analysis of other TCGA databases revealed that the expression of the signature genes also participate in the development of other tumor types (**Figure S3**). Our findings notwithstanding, the clinical application of the novel genes as biomarkers for diagnosis and prognosis of PCa need further validation.

## Conclusion

Overall, we identified a set of novel genes useful in accurate diagnosis and prognosis prediction of PCa. These findings set the foundation for the development of better and more effective markers for various PCa parameters. Therefore, further experimental and functional studies utilizing large samples are required to further validate the utility of the proposed biomarkers in the diagnosis and prognosis prediction of PCa.

## Supplementary Material

Supplementary figures and tables.

## Figures and Tables

**Table 1:** Characteristics of the GEO datasets

GEOset ID	Contributors	Platform ID	Samples	Number of rows per platform
GSE3325	Varambally S, et al	GPL570	6N 13T	54675
GSE6956	Wallace TA, et al	GPL1571	20N 69T	22277
GSE32448	Derosa CA, et al	GPL570	40N 40T	54675
GSE32571	Kuner R, et al	GPL6947	39N 59T	48652
GSE46602	Mortensen MM, et al	GPL570	14N 36T	54675
GSE55945	Arredouani MS, et al	GPL570	8N 13T	54675
GSE34312	Ashida S, et al	GPL6884	10N 10T	48803
GSE69223	Meller S, et al	GPL570	15N 15T	54675
GSE71016	Zhang L, et al	GPL16699	47N 48T	62976
GSE88808	Ding Y, et al	GPL22571	49N 49T	20260

Abbreviations: GEO: Gene Expression Omnibus; T: tumor samples; N: paracancerous normal samples.

**Table 2:** Visualization of specificity, sensitivity, and cutoff values of these 5 gene signatures as diagnostic and prognostic markers

Clinical traits	Risk score		χ^2^	*P* value
	High risk, n (%)	Low risk, n (%)		
**Age**			20.05	<0.0001
≤60	86 (38.74%)	136 (61.26%)		
>60	161 (58.97%)	112 (41.03%)		
**T**			8.085	0.0443
T1	82 (45.30%)	99 (54.70%)		
T2	98 (48.52%)	104 (51.48%)		
T3	64 (58.72%)	45 (41.28%)		
T4	3 (100.00%)	0 (0.00%)		
**N**			18.10	<0.0001
N0	173 (44.82%)	213 (55.18%)		
N1	74 (67.89%)	35 (32.11%)		
**M**			0.5165	0.4724
M0	242 (49.69%)	245 (50.31%)		
M1	5 (62.50%)	3 (37.50%)		
**Gleason Score**			75.88	<0.001
6	16 (35.56%)	29 (64.44%)		
7	82 (33.33%)	164 (66.67%)		
8	42 (66.67%)	21 (33.33%)		
9	104 (75.91%)	33 (24.09%)		
10	3 (75.00%)	1 (25.00%)		
**New tumor event**			41.70	<0.001
No	154 (41.51%)	217 (58.49%)		
Yes	93 (75.00%)	31 (25.00%)		

**Table 3:** Baseline characteristics of patients in TCGA cohorts

Gene	AUC	Std. Error	95% CI	Cutoff (log2)	Sensitivity %	Specificity %
ZNF695	0.7868	0.03956	0.7093 ~ 0.8643	3.566	67.8	84.62
CNEPA	0.8740	0.03153	0.8122 ~ 0.9358	4.413	81.53	86.54
TROAP	0.8923	0.02976	0.8340 ~ 0.9506	5.499	89.36	86.29
BIRC5	0.8864	0.02865	0.8302 ~ 0.9425	6.930	85.4	84.62
KIF20A	0.8506	0.03188	0.7881 ~ 0.9130	3.349	83.73	75.00

## Abbreviations

GEO: Gene expression omnibus database
TCGA: The Cancer Genome Atlas
DEGs: Differentially expressed genes
AUC: Area Under roc Curve
CI: Confidence Interval
RRA: Robust Rank Aggregation
WGCNA: Weighted Gene Co-expression Network Analysis
DFI: Disease-Free Interval
MSigDB: Molecular signatures database
GSEA: Gene set enrichment analysis
