# Supplementary material for: Five-gene signature associating with Gleason score serve as novel biomarkers for identifying early recurring events and contributing to early diagnosis for Prostate Adenocarcinoma
Source: J Cancer. 2021 Apr 30;12(12):3626–47. doi: 10.7150/jca.52170 (PMC8120165; doi:10.7150/jca.52170)
Supplement: Supplementary file 1 — Supplementary figures and tables. [file jcav12p3626s1.pdf]

Table S1. The primer sequences used to amplify the 5 genes.

Gene Symbol	Forward primer (5'->3')	Reverse primer (5'->3')	Product length (bp)
**ZNF695**	ACAGCCAGACACTCAGTTTTGT	TCCACTCACCCACAATTTCCC	160
**CENPA**	TAGGCGCTTCCTCCCATCAA	CACATATTTCTCTTGCCAGGCG	129
**TORAP**	TGTCCATCACCCTTTGGACG	GGATACCGATGCGCCAGG	214
**BIRC5**	TGACGACCCCATAGAGGAAC	CGCACTTTCTCCGCAGTTTC	186
**KIF20A**	GTGGCACGTCTTCGGACCTA	TTCTTGCGTACCACAGACCC	149

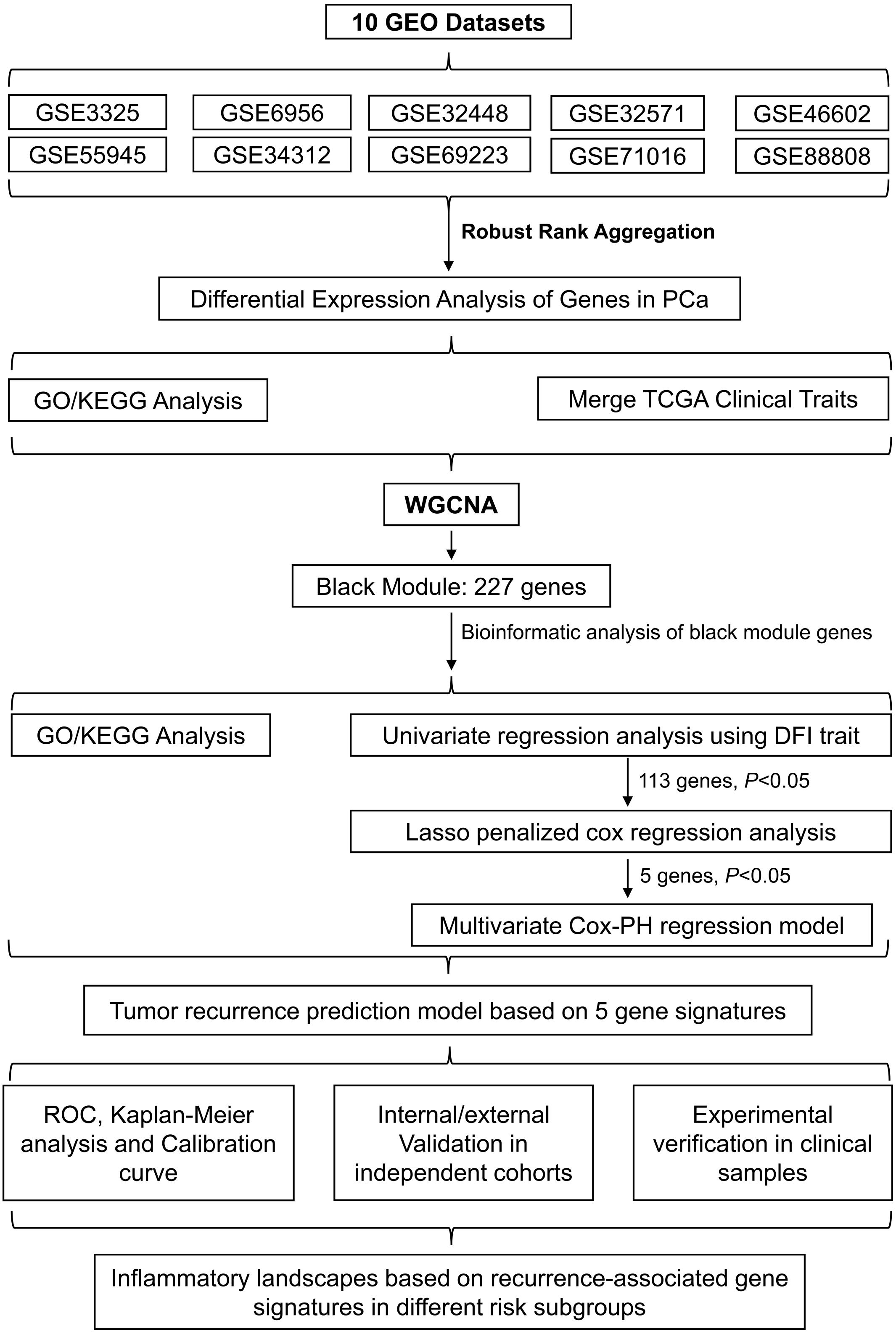

Figure S1. The flow chart of the analysis procedure.

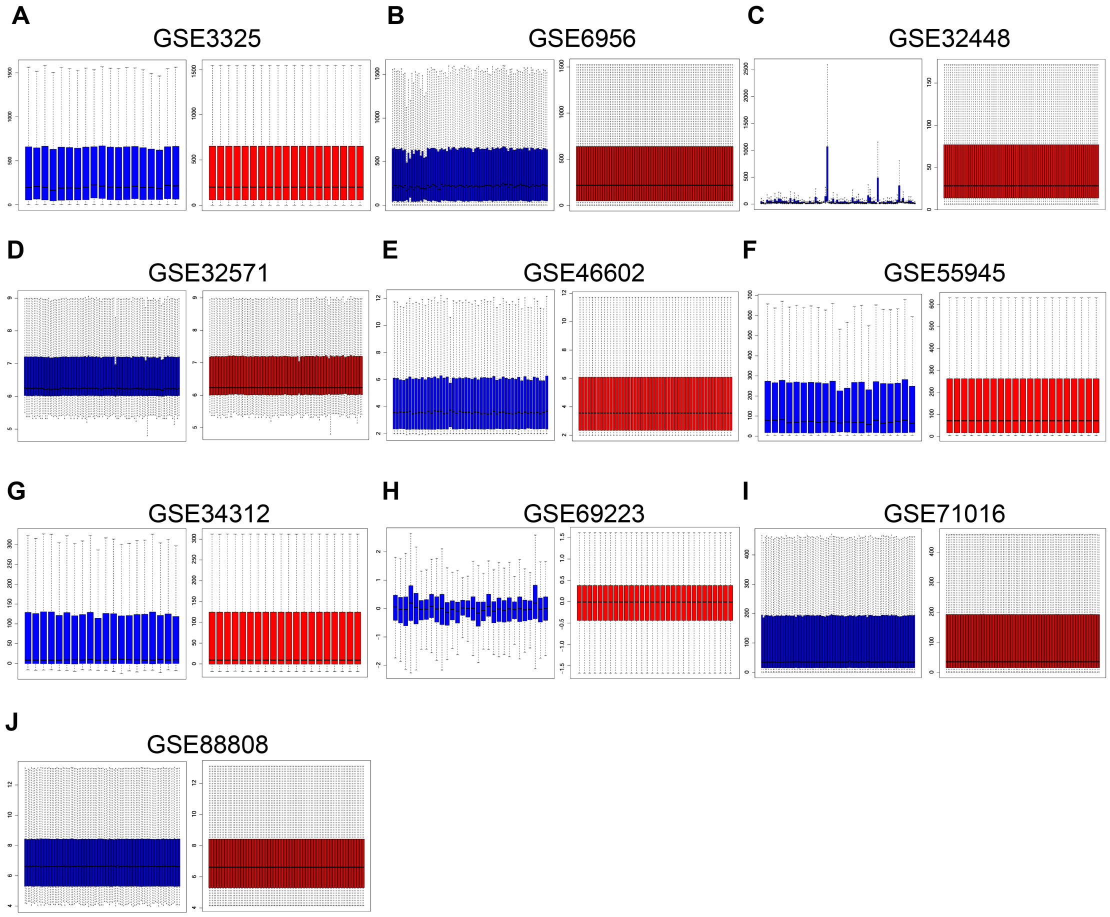

Figure S2. (A-J) RawBox graph shows normalized processing on GEO datasets.

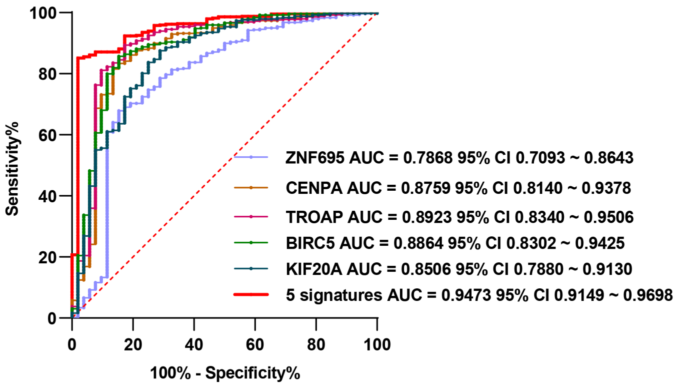

Figure S3. The combined diagnosis model based on 5 gene signatures shows higher diagnosis efficiency in TCGA cohort.

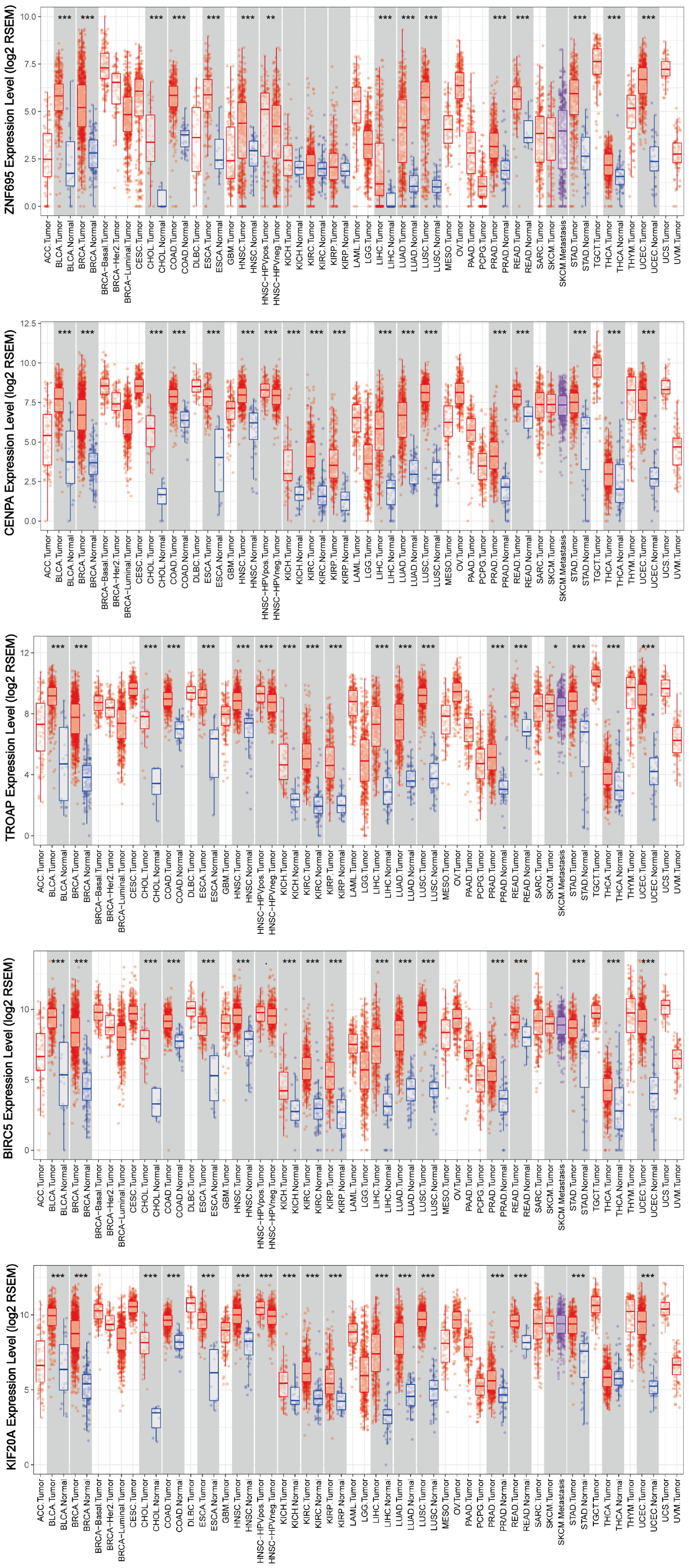

Figure S4. Assessment the imbalance of these 5 gene signatures in other tumors based on TCGA database algorithm.
